# Development of a Maize Molecular Evolutionary Genomic Database

**DOI:** 10.1002/cfg.282

**Published:** 2003-04

**Authors:** Chunguang Du, Edward Buckler, Spencer Muse

**Affiliations:** 1 Department of Biology and Molecular Biology Montclair State University Upper Montclair NJ 07043 USA; 2 US Department of Agriculture/Agricultural Research Service Raleigh NC 27695-7614 USA; 3 Department of Genetics North Carolina State University Raleigh NC 27695-7614 USA; 4 Bioinformatics Research Center North Carolina State University Raleigh NC 27695-7566 USA

## Abstract

PANZEA is the first public database for studying maize genomic diversity. It was initiated as a repository of genomic diversity for an NSF Plant Genome project on ‘Maize Evolutionary Genomics’. PANZEA is hosted at the Bioinformatics Research Center, North Carolina State University, and is open to the public (http://statgen.ncsu.edu/panzea). PANZEA is designed to capture the interrelationships between germplasm, molecular diversity, phenotypic diversity and genome structure. It has the ability to store, integrate and visualize DNA sequence, enzymatic, SSR (simple sequence repeat) marker, germplasm and phenotypic data. The relational data model is selected and implemented in Oracle. An automated DNA sequence data submission tool has been created that allows project researchers to remotely submit their DNA sequence data directly to PANZEA. On-line database search forms and reports have been created to allow users to search or download germplasm, DNA sequence, gene/locus data and much more, directly from the web.


**Comparative and Functional Genomics**
*Comp Funct Genom* 2003; **4**: 246–249.
Published online 1 April 2003 in Wiley InterScience (www.interscience.wiley.com). **DOI:** 10.1002/cfg.282

**Conference Review**

### Development of a maize molecular evolutionary genomic database

Chunguang Du,[1]* Edward Buckler[2,3] and Spencer Muse[4]

[1] *Department of Biology and Molecular Biology, Montclair State University, Upper Montclair, NJ 07043, USA*
[2] *US Department of Agriculture/Agricultural Research Service, Raleigh, NC 27695-7614, USA*
[3] *Department of Genetics, North Carolina State University, Raleigh, NC 27695-7614, USA*
[4] *Bioinformatics Research Center, North Carolina State University, Raleigh, NC 27695-7566, USA*

**Correspondence to: Chunguang Du, Department of Biology and Molecular Biology, Montclair State University, Upper Montclair, NJ 07043, USA. E-mail: duc@mail.montclair.edu*

Received: 4 February 2003
Revised: 7 February 2003
Accepted: 10 February 2003

### Abstract

**PANZEA is the first public database for studying maize genomic diversity. It was initiated as a repository of genomic diversity for an NSF Plant Genome project on 'Maize Evolutionary Genomics'. PANZEA is hosted at the Bioinformatics Research Center, North Carolina State University, and is open to the public (http://statgen.ncsu.edu/panzea). PANZEA is designed to capture the interrelationships between germplasm, molecular diversity, phenotypic diversity and genome structure. It has the ability to store, integrate and visualize DNA sequence, enzymatic, SSR (simple sequence repeat) marker, germplasm and phenotypic data. The relational data model is selected and implemented in Oracle. An automated DNA sequence data submission tool has been created that allows project researchers to remotely submit their DNA sequence data directly to PANZEA. On-line database search forms and reports have been created to allow users to search or download germplasm, DNA sequence, gene/locus data and much more, directly from the web.**
**Copyright © 2003 John Wiley & Sons, Ltd.**

**Keywords:** **database; maize; genome; evolution; germplasm; phenotype; SSR; DNA sequence**

### Introduction

Our overall objective is to design and implement a distributed information framework that will provide services, tools and infrastructure for high-quality analysis and annotation of large amounts of maize genomic diversity data. PANZEA (**http://statgen.ncsu.edu/panzea**) was initiated as a repository of genomic diversity for an NSF Plant Genome project on 'Maize Evolutionary Genomics'. More than 100 SSRs were screened for a comprehensive set of lines and accessions representing the maize germplasm pool, to characterize the amount and distribution of genetic diversity in maize germplasm, and to determine the degree of genetic similarity among different segments of the germplasm pool. DNA sequences of 80–100 loci along chromosomes 1 and 3 were generated for 15 representative maize lines, to establish the relationship between recombination rates, natural selection and genetic diversity, and to examine the degree of linkage disequilibrium among and within loci.

The DNA sequences of 19 candidate genes were produced for 100 inbred lines. Phenotypic data on agronomic traits controlled by these candidates were collected for this same set of inbreds to measure associations between nucleotide polymorphisms in the candidate genes and the phenotypic traits they affect.

PANZEA is hosted at the Bioinformatics Research Center, North Carolina State University, and is open to the public. The PANZEA database represents a public repository of information on molecular evolutionary genomics of maize. Knowing what the various data mean, and the biological information that can be obtained from these data,

Copyright © 2003 John Wiley & Sons, Ltd.

greatly advances our understanding of maize evolutionary genomics. New computational methods for analysing maize evolutionary genomics are dependent on having a complete maize genomic database.

### Method

Oracle8i was used as the PANZEA database management system. Oracle was installed on a Sun Ultra 60 workstation as the database server. We are running a Solaris 8 UNIX operating system and the Apache web server. The automated data submission tool was developed with JBuilder (**http://www.borland.com/jbuilder**) and ColdFusion (**http://www.macromedia.com**) was used to generate part of the on-line search forms.

### Database design

We chose the relational data model for our representation of data from the evolutionary genomics of maize project. The relational model has the advantage of providing a natural view of the structure of the data system. The PANZEA database was implemented with Oracle8i, providing efficient, reliable, secure data management for high-end applications. The PANZEA database is a structured communication channel, through which members of the PANZEA project distribute the results of their work to their peers. The researchers communicate directly with the database and are responsible for ensuring the accuracy of the data. The database is focused around germplasm and loci and performs well at relating diverse germplasms with phenotypic, sequence and polymorphism data. Figure 1 shows the core table of the database schema. There are three types of database set up for PANZEA, the production database, the public database and the test database.

The germplasm and passport data are separated into different tables. This separation has several merits. The seed lot and plant number information are entered into the germplasm table, which allows users to track the single plant that they have sampled. The 'stockid' in the germplasm table is the main primary key, and links with the phenotype, polymorphism and alignment tables. Most of the passport data are static and are kept in one table, making updating the germplasm table

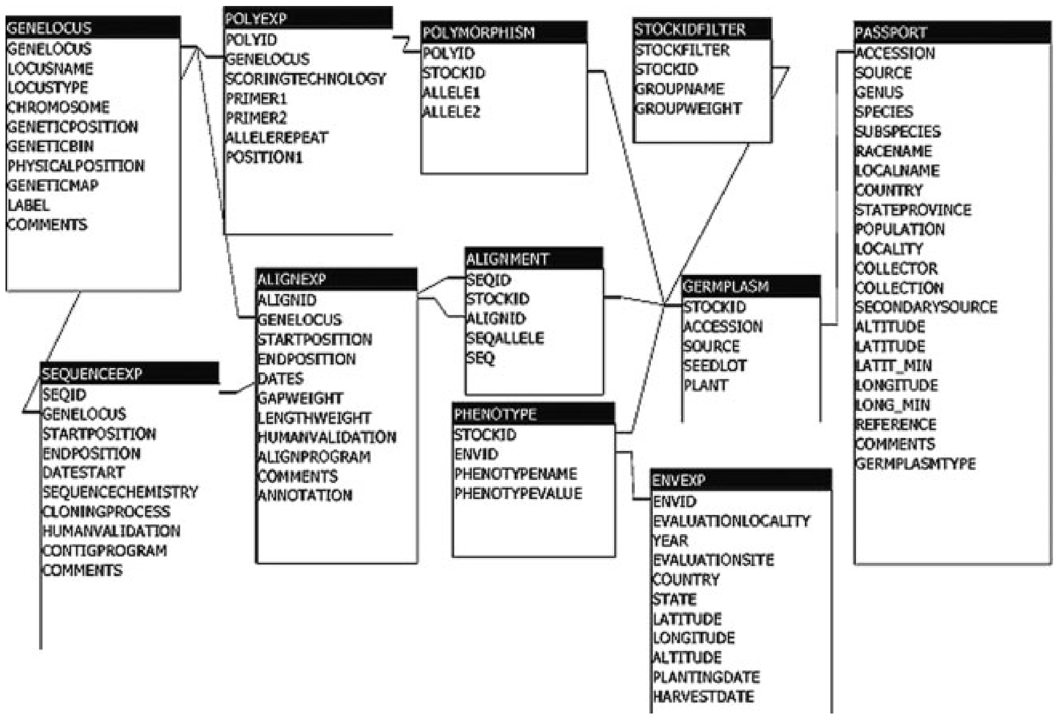

**Figure 1.** The PANZEA schema

Copyright © 2003 John Wiley & Sons, Ltd.

simple and easy. DNA sequence and molecular markers are the two major types of data collected in this project; separated tables are used to capture these two kinds of data. The polymorphism table can store SSRs, SNPs (single nucleotide polymorphisms) and other types of markers. Many different types of phenotypic data will be collected as the project moves on, so, rather than placing phenotype names statically into the phenotype table, we have made the phenotype table update the phenotype name dynamically. This avoids the need to add columns into the phenotype table as new phenotypes are determined.

### Automated data submission

An automated DNA sequence data submission tool has been created (Figure 2). This allows project

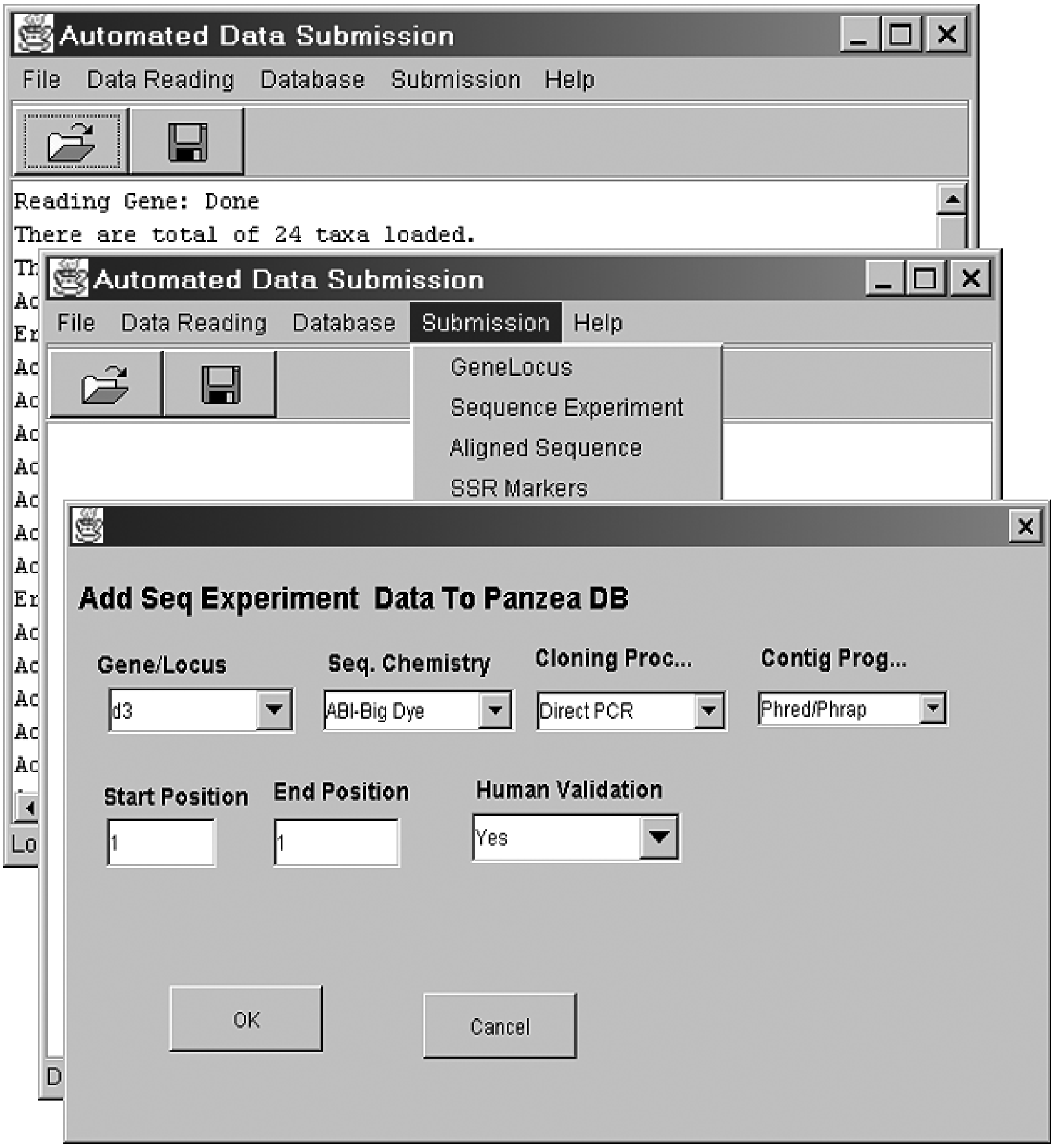

**Figure 2.** The automated data submission tool

Copyright © 2003 John Wiley & Sons, Ltd. 

researchers to remotely submit their DNA sequence data directly to the PANZEA database. Project personnel can submit their sequence, SSR and phenotype data to the PANZEA database from their local machine. The program can read NEXUS, tab delimited text files, and SSR data generated by Celera. It is possible to upload data related to gene locus, sequence experiment, aligned sequence, SSR marker allele frequencies and phenotypic data.

### Data in the production database

- *Passport data*: There are over 2000 accessions with passport data on seed source and location; about 80% of these are maize germplasm, 281 are inbred lines and 182 are teosinte.
- *SSR data*: There are close to 100 000 data points from diverse germplasms, most of these are inbred lines and teosinte.
- *Aligned sequences data*: 8000 sequences from 100 genes have been aligned.
- *Phenotype data*: There are 15 000 data points in the phenotype table so far. These correspond to aerial mass, biomass, cob diameter, cob weight, days to pollen, days to silk, ear diameter, ear height, ear length, ear mass and others. There will be a large amount of DNA sequences and phenotype data uploaded to the database in the very near future.

### Web query interface

On-line database search forms and reports have been created for the collaborators in this project to use. They can search or download germplasm, DNA sequence, genelocus data, and much more, directly from the web. Moderately complex search forms are also available on the web for the project collaborators and the public.

- *User interface*: dynamic HTML (DHTML) was used to design the Web interface for the database front-end. DHTML includes any combination of HTML, JavaScript, Cascading Style Sheets (CSS) and the Document Object Model (DOM). The interface is composed of reports and forms.
- *Middle tier*: Application server with statistical analysis tools.
- *Overall architecture*: database (backend server) $\leftrightarrow$ middleware $\leftrightarrow$ web Interface.

### Refinement of the design

The PANZEA schema was designed at the beginning of the project, after consultation with the biologists working on the project. As the project developed, it became clear that there were several aligned sequences with the same 'alignid', 'seqid', and 'stockid'. To resolve these we added another composite key, 'seqallele', to uniquely identify aligned sequences. This is just one example of the modifications that have been made since the initial design.

### Future plans

Over time, the schema will inevitably alter. In particular we wish to accommodate multiple alignment approaches, recording of phenotype acquisition methods, and the inclusion of tetraploids.

### Acknowledgements

We thank Major Goodman, John Dobley, Bruce Weir and Brandon Gaut for their help in identifying the requirements for the database design. We acknowledge the help of Mary Polacco's group from MaizeDB for initial discussions on the PANZEA schema. This database was supported by National Science Foundation Grant DBI-0096033.

Copyright © 2003 John Wiley & Sons, Ltd. 

